# The use of Edinburgh Postnatal Depression Scale to identify postnatal depression symptoms at well child visit

**DOI:** 10.1186/1824-7288-35-32

**Published:** 2009-10-28

**Authors:** Vincenzo Currò, Emilia De Rosa, Silvia Maulucci, Maria Lucia Maulucci, Maria Teresa Silvestri, Annaluce Zambrano, Vincenza Regine

**Affiliations:** 1 Department of Pediatrics, Catholic University, Rome, Italy; 2 Department of Psychiatry, Catholic University, Rome, Italy; 3 Istituto Superiore di Sanità, Rome, Italy

## Abstract

**Objectives:**

1) to evaluate the role of the pediatrician in detecting postnatal depression (PD) symptoms by the Edinburgh Postnatal Depression Scale (EPDS); 2) to detect factors increasing the risk of PD and, 3) to assess the importance of scores gained from fathers' questionnaire.

**Methods:**

we surveyed 1122 mothers and 499 fathers who were assessed using the EPDS during the first well-child visit. After 5 weeks, high scoring parents, completed a second EPDS. High scoring parents were examined by a psychiatrist who had to confirm the PD diagnosis.

**Results:**

26.6% of mothers and 12.6% of fathers at the first visit, 19.0% of mothers and 9.1% of fathers at the second visit, gained scores signaling the risk of PD. Four mothers and two fathers had confirmed PD diagnosis. Younger maternal age, non-Italian nationality and low socio-economic condition were related to higher EPDS scores.

**Conclusion:**

PD is common in the average population. Using a simple and standardized instrument, pediatricians are able to detect parents with higher risk of suffering from PD.

## Background

Postnatal depression (PD) is the most common disorder following childbirth and a social problem for public welfare: ten to fifteen women out of one hundred suffer from this disorder [1]. PD is a severe condition that has been described as "a thief who steals maternity"; up to 50% of the cases are diagnosed, and approximately 49% of women who seek help feel desperately depressed [2]. If untreated, a large number of these mothers continue to be depressed until the end of the first and the second postnatal years.

Women who suffer from PD are exposed to an increased risk of future depression, relapses, thoughts of abusing their children, and face difficulties in the child-mother relationship [3]. Maternal Depression (MD) may have a strong negative impact on the social, cognitive and behavioral development of children, including an increased rate of behavioral problems at school [4,5]. The obstetrician should be the first to identify those mothers who risk developing depression, but this can be quite difficult as symptoms often appear after the routine 4 to 6 week postnatal examination. Moreover, mothers with PD often do not recognize the symptoms of depression. This is a result of the difficulty in identifying the following signs as symptoms of PD: weight loss, irritability, crying fits and fatigue (often considered as the physiological adaptation to life with a newborn child). This is the reason why many women do not receive an immediate diagnosis or an appropriate treatment program [6]. Pediatricians may be the only medical workers that are routinely met by mothers during the first twelve months of the baby's life [7]. A self- report rating scale routinely administered in pediatrics could be a useful tool to recognize the risk of PD. The Edinburgh Postnatal Depression Scale is the most widely used screening scale for PD [8]. It has been validated in Holland, Australia, Portugal, Sweden, Italy, Spain, United Arab Emirates, France and India [6,8-15].

The EPDS has already been administered in a pediatrics setting at the University Rochester Medical Centre (NY), although the impact of this screening instrument on the visit time was not assessed. However, the length of the visit is an important factor to take into consideration when working in a busy pediatric clinic. As a matter of fact, if we extend visit times, we risk reducing the total number of daily visits.

In this research project we focused on PD risk in mothers who have recently given birth. Many studies highlighted that the most significant risk factors for PD are: young maternal age, absence of a social support, immigration, and lack of a supporting spouse [16]. In addition, we also took into account what the outcome would be if new fathers were evaluated as well. To date, only a few studies have investigated PD risk in fathers, and no study has been conducted in Italy. Men who have manual or working class occupations and low social integration are more likely to become depressed [9]. In this study we will refer to parents at both low and high risk of suffering from depression on the basis of the EPDS score. In fact, a high EPDS score does not mean postnatal depression but only a high risk of suffering from PD. A psychiatrist was involved to confirm the diagnosis of PD. The aims of our study were: 1) to check the feasibility of assessing the risk of PD in parents using the EPDS; 2) to provide correlations between PD risk and socio-demographic information, both in mothers and in fathers; 3) to check correlations between high EPDS scoring fathers in couples with EPDS positive mothers and high EPDS scoring fathers in couples with EPDS negative mothers.

## Methods

### Setting

The study was conducted at the Pediatric Clinic of the Policlinico A. Gemelli, Catholic University Hospital, Rome.

This clinic admits 4000 patients a year, and it is a teaching site for pediatric residents and medical students.

### Participants

Our team included: a senior pediatrician, two pediatric residents, two psychologists, and two psychiatrists. The EPDS was proposed to all parents, regardless of age and nationality, at the first postnatal check-up within the first year of baby's birth. Unmarried women were also included. Postnatal check-up examinations were carried out by the senior pediatrician with the aid of pediatric residents.

### Research Tools

EPDS is a paper-and-pencil self-reporting questionnaire composed of 10 questions and a 0-3 point scale. The cut-off score is 9 for women and 7 for men [11,17,18].

### Procedure

EPDS was proposed by a pediatrician, before clinical examination. The informed consent was obtained by explaining the meaning of the EPDS and we asked participants to complete questionnaires, without any help, and to answer according to their feelings during the previous seven days. In the case of high scores, the EPDS was repeated after five weeks, especially when we visited babies in the first 15 days of life, as, after delivery, many women experience 'baby blues'. Baby blues is considered a normal stage of early motherhood, usually disappearing some days after delivery.

The EPDS was translated and validated into several languages (Italian, French, Spanish, English, Arabic, and Punjabi) for parents who did not understand Italian. We translated the EPDS into Singhalese and used it, although this version was not officially validated.

Whenever our test results were rated as 'high', we told mothers or fathers or sometimes both parents, to undergo a psychologist-psychiatrist examination. Without an appropriate psychiatric evaluation, a high EPDS score does not mean PD. Sometimes, in the self-reporting test, people report anxiety, mood instability, depressed mood, and other transient emotional disturbances which disappear within a few hours or days. A PD diagnosis requires a woman to be experiencing dysphoric mood and several other symptoms such as appetite, sleep or psychomotor disturbances, excessive feeling of guilt, fatigue and suicidal thoughts for a minimum of two weeks.

### Study Population

This cross-sectional study started in January 2005 and ended in November of the same year. 1130 infants were examined at the first postnatal check-up (median 17 days, range 15-20 days). We proposed the EPDS to 1628 parents; 1621 subjects, 1122 mothers of the 1127 (99.6%) and 499 fathers of the 501 (99.6%) completed the EPDS and were included in this study. Five mothers with previous depression symptoms and two fathers who did not complete the EPDS were excluded. The male group was smaller than the female one, due to the fact that fathers were investigated only from August onwards and mothers often attended the clinic alone.

We excluded mothers with a history of depression since we intended to check only symptoms of PD, which is a perinatal pathology, not to be confused with depression.

### Statistical Analysis

To evaluate the feasibility of assessing postnatal depression symptoms, we calculated by how many minutes (average and range) the completion of the questionnaire extended the visit time, and the percentage of compilers.

The prevalence of the risk of PD was calculated as the percentage of mothers who scored ≥ 10 and as the percentage of fathers who scored ≥ 8. The characteristics of subjects at high PD risk and at low PD risk were compared using the Chi-squared test for each following characteristic: maternal and paternal age, marital status, employment, educational level, nationality, nursing, number of pregnancies, gestational age, delivery, mother's and father's pathologies, baby's weight at birth, gender, infant hospitalization and pathologies, and the season of the interview. Crude odds ratios (OR) were also calculated for all variables and adjusted OR were also calculated for variables where the univariate analysis showed a statistically significant association (p-value for Chi squared test < 0.05). Adjusted OR were calculated with the construction of a multivariate logistic regression model using the backward elimination method. The fit of the model was assessed using the Hosmer-Lemenshow test.

## Results

### Length Of The Visit

The time range taken to complete the test was 2-7 minutes, with a mean of 3.28 minutes for women and 3.22 minutes for men. Less time was employed if parents had no problems with the language: 2.98 minutes (range: 2-4) for Italian females and 2.98 minutes (range: 2-5) for Italian males; 5.1 minutes (range: 2-7) and 5.6 minutes (range: 3-7) for foreign females and males, respectively. For foreigners who did not have the text translated into their mother tongue, the time was longer: 6.00 minutes (range: 5-7) for both females and males. For foreigners with the test translated into their mother tongue, the time was 3.73 minutes (range: 2-6) for females and 4.33 minutes (range: 3-5) for males.

### Socio-Demographic Characteristics of the Sample

#### Newborns

Table 1 shows the demographic and clinical characteristics of the 1,130 infants: 51.1% were male, 79.8% were Italian, 62.6% were first-born, 25.3% had pathologies and 2.1% were hospitalized.

**Table 1:** Demographic and clinical characteristics of 1,130 infants

	**Number**	**Percentage**
**Sex**		
Male	577	51.1
Female	553	48.9
Nationality*		
Italian	896	79.8
Non Italian	227	20.2
**Brothers**		
No	707	62.6
Yes	423	37.4
**Twin birth**		
No	1127	99.7
Yes	3	0.3
Weight at birth*		
≤ 2500 g	64	5.7
> 2500 g	1064	94.3
Gestational Age*		
≤ 36 wk	74	6.6
> 36 wk	1052	93.4
Nursing*		
Breastfeeding	756	69
Bottlefeeding	115	10.5
Both	224	20.5
**Hospitalization**		
No	1106	97.9
Yes	24	2.1
**Pathology**		
No	844	74.7
Yes	286	25.3

*Information is not available for all infants

As "pathology", we considered any problems, even not serious, signed in the discharge papers of the newborn babies that could create anxiety to parents (e.g. jaundice, hip instability, cefaloematoma, hypocalcemia, patent foramen ovale).

Hospitalization regarded surgery, phototherapy, infections, prematurity, respiratory distress syndrome (RDS).

#### Mothers

The mean age of the 1,122 mothers assessed was 32.9 years (SD 4.9), 62.7% were primiparae with a mean age of 31.7 years, 96.9% were married or lived with their partner and 20.2% were non-Italian. 85.1% had a school diploma or degree, 72.9% were employed; 713 women (63.6%) had natural delivery, 409 (36.4%) had a caesarean delivery, and 49 women had previous spontaneous abortion.

Countries of origin of the 223 non-Italian mothers were: 43.5% East Europe, 25.7% South America, 14.1% Asia, 10.5% West Europe and North America, and 6.2% Africa.

#### Fathers

The mean age of the 499 fathers assessed was 36.3 years (SD 5.5); 99.6% were married or lived with their partner; 10.1% were non-Italian; 79.7% had a school diploma or degree and 98.6% were employed.

Countries of origin of the 50 non-Italian fathers were: 32.0% East Europe, 24.0% South America, 20.0% Asia, 12% West Europe and North America, 12.0% Africa.

### Morbidity

#### Mothers' morbidity

Interviewed mothers had an EPDS mean score of 7.11 (SD 4.42). 298 (26.6%) mothers had an EPDS score ≥ 10 and 824 (73.4%) had an EPDS score <10. For those mothers with a low depression risk the mean score was 4.98 (SD 2.55), whereas for mothers with a high risk of depression, the mean score was 13.01 (SD 2.88).

#### Fathers' morbidity

The total EPDS mean score was 3.86 (SD 3.12). 63 fathers had an EPDS score ≥ 8 with a 12.6% risk of depression. The mean score for 63 high risk fathers was 9.98 (SD 2.11) and 2.97 (SD 2.07) for 436 low risk fathers.

### Correlations Between the Risk of Pd and Socio-Demographic Information

#### Mothers

Table 2 shows the demographic and clinical characteristics of both high and low depression risk mothers, the occurrence of PD risk, crude OR (COR) and adjusted OR (AOR).

**Table 2:** Demographic and clinical characteristics of high PD risk mothers vs low PD risk mothers

	**Mothers at high risk of PD** **(n = 298)**	**Mothers at low risk of PD** **(n = 824)**	**Prevalence** **%**	p-value**	**Crude OR** **(95% CI)**	**Adjusted OR** **(95% CI)**
Age group*						
Mean age (SD)	31.95 (5.60)	33.23 (4.54)		0		
< 25 years	35	25	58.3		4.25 (2.50-7.23)	3.12 (1.88-5.82)
≥ 25 years	263	798	24.8		1	1

Nationality*						
Italian	205	687	23	0	1	1
Non Italian	92	131	41.3		2.35 (1.73-3.20)	2.01 (1.43-2.82)

Marital status						
Married^	283	805	26	0.019	1	1
Single	15	19	44.1		2.25 (1.13-4.48)	1.49 (0.69-3.19)

Educational level*						
Primary	6	4	60	0.001	4.58 (1.28-16.38)	2.30 (0.58-9.12)
Middle	55	98	35.9		1.71 (1.19-2.46)	1.34 (0.90-1.99)
High^+^	234	715	24.7		1	1

Number of pregnancies*						
1	209	494	29.7	0.002	1.56 (1.17-2.07)	1.33 (0.98-1.80)
≥ 2	89	328	21.3		1	1

Feeding of infant*						
Breastfeeding	178	573	23.7	0.003	1	1
Bottlefeeding^#^	109	227	32.4		1.54 (1.16-2.04)	1.67 (1.20-2.17)

Employment*						
No	94	209	31	0.097	1.28 (0.95-1.72)	
Yes	213	605	26		1	

Pathology of mother						
No	164	473	25.7	0.479	1	
Yes	134	351	27.6		1.10 (0.84-1.44)	

Pathology of father*						
No	85	250	25.4	0.988	1	
Yes	41	121	25.3		1.00 (0.63-1.57)	

Gestational Age*						
≤ 36 wk	19	55	25.7	0.872	0.96 (0.54-1.69)	
> 36 wk	277	767	26.5		1	

Delivery*						
Vaginal	173	535	24.4	0.058	1	
Abdominal	120	285	29.6		1.30 (0.99-1.71)	

Gender of infant						
Male	149	421	26.1	0.747	1	
Female	149	403	27		1.04 (0.79-1.37)	

Weight at birth*						
≤ 2500 g	19	45	29.7	0.543	1.19 (0.68-2.06)	
> 2500 g	277	779	26.2		1	

Pathology of infant						
No	217	621	25.9	0.687	1	
Yes	77	207	27.2		1.06 (0.78-1.46)	

Hospitalization of infant						
No	286	814	26	0.893	1	
Yes	6	16	28.6		1.07 (0.37-2.93)	

Season of interview*						
Autumn	53	176	23.1	0.568	1	
Winter	57	164	25.8		1.15 (0.75-1.77)	
Spring	100	254	28.2		1.31 (0.89-1.92)	
Summer	86	230	27.2		1.24 (0.84-1.84)	

Note: Variables with a p-value < 0.05 were included in the multivariate analysis

*Information is not available for all mothers

** p-value for Chi-square test

^include common-law wife

^+^include diploma and degree

^#^include mixed breastfeeding

The occurrence of a high risk of PD is significantly higher (p < 0.05) among <25 years of age vs. ≥ 25 years of age (58.3% vs. 24.8%); among non-Italians vs. Italians (41.3% vs. 23.0%); among singles compared to married women (44.1% vs. 26.0%); among those with a lower level of education (60.0% and 35.9%) compared to those with a higher level of education (24.7%); among primiparae vs. multiparae (29.7% vs. 21.3%) and among bottle-feeding mothers vs. breast-feeding mothers (32.4% vs. 23.7%). The multivariate logistical regression model shows that mothers with high depression risk were: <25 years of age (AOR, Adjusted Odds Ratio, 3.12; 95%; CI 1.88-5.82), non-Italian (AOR 2.01, 95%; CI 1.43-2.82), bottle-feeding (bottle-feeding vs. breast-feeding AOR = 1.67; CI 1.20-2.17). Marital status and educational level which appeared to be significant in the univariate analysis were no longer significant in the multivariate analysis. We did not find any significant association (p-value for Chi squared test p > 0.05) between PD risk and low birth weight babies, gender, newborn pathologies and hospitalization, gestational age, delivery, employment, maternal and paternal pathology, and seasonality. There were no significant differences among non-Italian mothers (p > 0.05): the occurrence of a high risk of PD was 45.8% for mothers born in East Europe, 36.7% in South America, 42.3% in Asia, and 58.3% in Africa.

#### Fathers

Table 3 shows the demographic and clinical characteristics of both high risk and low risk fathers, the prevalence of father risk of suffering from depression, COR and AOR. The occurrence of the risk of paternal depression was significantly higher (p < 0.01) among <30 years of age vs. ≥ 30 years of age (27.3% vs. 11.2%); among non-Italians vs. Italians (24.0% vs. 11.3%); among those with a lower level of education (primary or middle education 21.8% vs. high education 10.3%); among unemployed or unskilled employment vs. other employment (30.2% vs. 10.4%).

**Table 3:** Demographic and clinical characteristics of high PD risk fathers vs low PD risk fathers

	**Fathers at high risk of PD** **(n = 63)**	**Fathers at low risk of PD** **(n = 436)**	**Prevalence** **%**	p-value**	**Crude OR** **(95% CI)**	**Adjusted OR** **(95% CI)**
Age group						
Mean age (SD)	35.32 (6.72)	36.41 (5.34)		0.002		
< 30 years	12	32	27.3		2.97 (1.44-6.13)	1.98 (0.86-4.61)
≥ 30 years	51	404	11.2		1	1

Nationality*						
Italian	50	394	11.3	0.01	1	1
Non Italian	12	38	24		2.49 (1.22-5.07)	1.23 (0.51-2.95)

Educational level*						
Middle^+^	22	79	21.8	0.002	2.42 (1.36-4.29)	1.71 (0.92-3.20)
High^++^	41	356	10.3		1	1

Employment*						
Unskilled°	16	37	30.2	0	3.73 (1.93-7.23)	2.24 (1.02-4.96)
Skilled	46	397	10.4		1	1

Marital status*						
Married^	62	434	12.5	0.111	1	
Single	1	1	50		7.0 (0.43-113.35)	

Pathology of father						
No	42	293	12.5	0.933	1	
Yes	21	143	12.8		1.02 (0.58-1.79)	

Pathology of mother						
No	43	259	14.2	0.179	1	
Yes	20	177	10.2		0.68 (0.37-1.24)	

Number of sons*						
1	41	272	13.1	0.745	1.10 (0.61-1.98)	
≥ 2	22	160	12.1		1	

Gestational Age						
≤ 36 wk	4	25	13.8	0.845	1.11 (0.37-3.32)	
> 36 wk	59	411	12.6		1	

Delivery*						
Vaginal	39	281	12.2	0.777	1	
Abdominal	23	153	13.1		1.08 (0.62-1.88)	

Gender of infant						
Male	37	221	14.3	0.232	1	
Female	26	215	10.8		0.72 (0.41-1.27)	

Weight at birth						
≤ 2500 g	4	24	14.3	0.785	1.16 (0.39-3.47)	
> 2500 g	59	412	12.5		1	

Pathology of infant						
No	42	307	12	0.231	1	
Yes	24	126	16		1.39 (0.78-2.47)	

Hospitalization of infant						
No	58	421	12.1	0.699	1	
Yes	3	17	15		1.28 (0.29-4.83)	

Feeding of infant*						
Breastfeeding	33	274	10.7	0.205	1	
Bottlefeeding^#^	25	145	14.7		1.43 (0.79-2.59)	

Season of interview*						
Autumn	25	201	11.1	0.555	1	
Winter	0	7	0		-	
Spring	6	32	15.8		1.51 (0.57-3.96)	
Summer	31	196	13.7		1.27 (0.72-2.23)	

Note: Variables with a p-value < 0.05 were included in the multivariate analysis

*Information is not available for all fathers

** p-value for Chi-square test

^+ ^include primary education

^++^include diploma and degree

°include unemployed

^include common-law husband

^# ^include mixed breastfeeding

The multivariate logistic regression model shows that employment was only associated with depression risk among fathers; in particular, the risk of depression was double (AOR 2.24; 95% CI 1.02-4.96) among unemployed or unskilled employed fathers vs. skilled employed fathers. Age, nationality and level of education which appeared to be significant in the univariate analysis were no longer significant in the multivariate analysis. We found no statistical association (p-value for Chi squared test p > 0.05) between paternal risk of suffering from depression and the other variables considered: marital status, number of sons, low birth weight babies, gender, type of feeding, newborn pathologies and hospitalization, gestational age, delivery, maternal and paternal pathology, and seasonality.

There were no significant differences among nationalities of non-Italian fathers (p-value > 0.05): the occurrence of a high risk of PD was 12.5% for fathers born in East Europe, 33.3% in South America, 40.0% in Asia, and 33.3% in Africa.

### A High Epds Score Must be Confirmed by a Psychiatrist

The psychiatrist was required to confirm the PD diagnosis in the 147 mothers and 22 fathers that attended the second visit and had scores signaling a risk of PD, 19.0% and 9.1% respectively. The psychiatric evaluation lowered the range of the EPDS positivity by 4 mothers and 2 fathers. The low number of parents with a diagnosis of PD did not permit to calculate the degree of association between the characteristics of the PD group and the factors found to be predictive of an increased risk for PD.

### Couple Morbidity

497 mothers were interviewed with their partner. This sample of mothers was composed of women who were: Italian (83.5%), married (99.4%), and skilled employees (23.4%). No statistical differences were found between the occurrence of the risk of depression in mothers with or without a partner (25.3% and 27.6% respectively).

The prevalence of couples who risked suffering from depression (risk in both parents: mother with EPDS ≥ 10, father with EPDS ≥ 8) was 6.2% (31 couples). 340 couples (68.4%) did not have high scores. We observed 126 cases with the risk of depression in one parent: 95 (19.1%) in mothers and 31 (6.2%) in fathers.

The percentage of fathers with EPDS ≥ 8 was significantly higher (p < 0.01) among fathers in couples with high risk mothers (24.6%) than among fathers in couples with low risk mothers (8.4%). The risk of depression in fathers of couples with high risk mothers was approximately four times higher (OR 3.6; 95% CI 2.1-6.2) than in those of couples with low risk mothers.

## Discussion

We carried out a pilot study in order to verify the possibility to use the EPDS and to detect its limitations. We analyzed the problems which emerged from the American study, such as the length of the visit [7]. Glaze and Cox performed a computerized version of psychiatric rating scales that may be less time-consuming than the pencil-and-paper method [19]. We have not used computerized versions of the test yet, but we are carrying out the self-reporting test during the anamnesis. Collecting data extended visit time from 1 to 2 minutes for foreign parents who did not have the EPDS translated into their native language. As to Italian parents, we did not notice any extension of the visit time. Thus, while the pediatrician collected information from papers (discharge form, nursery, vaccine book, etc.) and recorded them in a file in 5 minutes, parents could complete the test with a little effort to save time. Every time we introduce an innovative change into a well-established routine of a busy hospital we must be very careful not to increase visit times previously arranged with the directional management in order not to increase costs or cause a loss in profits. The high participation rate (99.6%) demonstrates that the EPDS is easy to understand.

Our study confirmed that the risk of PD is more common in foreign women. In London, Onozawa showed that women coming from ethnic minorities or from a non-English speaking background should be regarded as high risk group for postnatal depression [20]. Pregnancy, giving birth and bringing up a child, are also a psychological/cultural matter. We believe that environmental support and cultural models could fail in a migrant context, which is why immigrant mothers often live in a high psychic risk situation. In such critical situations we create a protective net not only medically but also socially, involving a welfare officer.

Results from our research show that fathers have lower depression rates than mothers [21,9]. However, the little number (two) of father with PD did not allow any conclusions. In our study the higher risk of paternal depression was associated with a lower level of employment. Moreover, work instability raises PD rate in fathers. Fathers give their family both a psychological and material support. If fathers do not feel capable of fulfilling this task, they could become depressed [22]. This could be one of the reasons why, in our society, males enter parenthood later than females.

In our study, the risk of PD was significantly associated with younger mothers (< 25 years of age) and the univariate analysis showed that younger fathers (< 30 years of age) were more frequently exposed to the risk of depression. Adolescents or young parents often perceive their child's birth as an obstacle to their identity [22]. Parents who are not able to fulfill their life projects or to adapt to the role of parents could become depressed. We also found that early diagnosis is important in parents with a high risk of PD. Prevention is necessary for those who are vulnerable, i.e., migrant or adolescent parents. Psychological and social support could decrease the risk of PD [23].

The risk of PD was lower in breast-feeding mothers than in bottle-feeding mothers. Can the absence of PD lead to an increased probability of breastfeeding or the breastfeeding is a protective factor against PD? [24]. Our results demonstrated an association between the two variables, independent from the other factors taken into account, and not a causal pathway in which breastfeeding "protects" against PD. The reverse can also be true: the absence of PD can lead to an increased probability of breastfeeding that perhaps is more plausible. A statistical association found in a multivariate analysis is not enough to demonstrate the presence of a causal relationship.

We found no difference in the occurrence of PD during different seasons [25].

Our results confirmed that the risk of depression for fathers in couples where the mother is depressed is approximately four times higher than that of fathers in couples with non-depressed mothers.

### Limitations Of This Study

A possible limitation of this study is that it was conducted in just one pediatric clinic, which was situated in an academic medical centre. Postnatal child assessment is made by family pediatricians in their private offices, where a team comprising psychologists and psychiatrists is not usually available. The extension to other practices, such as in pediatric family physician practices, would require further investigation. The feasibility of performing an evaluation with the EPDS in a well structured and trained setting does not mean that it can automatically be proposed for a more widespread and general use.

Moreover, the EPDS was used at each first well-child visit, even when the children were a few days old. It would be better to administer the EPDS from 40 days to 12 months of baby's birth, in order to check PD and overcome the risk of postnatal blues.

Another limitation is that we performed the second EPDS only on 147 mothers and 22 fathers, instead of on the 298 mothers and 63 fathers, with an high first EPDS. Therefore the high depression scores at the second visit needs to be evaluated with caution, due the high lost at the follow up. The question of whether EPDS scores or demographic characteristics at the first postnatal visit predict a diagnosis of PD would require further investigations.

Another limitation was that we did not have information regarding parent follow-up or treatment with the diagnosis of PD, thus limiting our ability to comment on the effectiveness of depression symptom detection.

We also did not examine the influence of depression on early breastfeeding termination [26]. However, breastfeeding difficulties and subsequent early breastfeeding termination may encourage postnatal depression symptoms to appear.

## Conclusion

The authors presented results of a personal experience in the setting of an outpatient pediatric clinic for newborn infants at a large and specialized teaching hospital. The aim of this study was to detect factors increasing the risk of PD in the mothers and the fathers using EPDS.

Mothers and fathers often experience a moment of crisis and loneliness which they are not able to discuss because of their new frenetic lifestyle. Sometimes such problems are reported in the media as a kind of tragedy.

To assess PD in parents is very important because psychological treatments improve very quickly maternal mood and mother-infant interaction [23].

## Abbreviations

EPDS: Edinburgh Postnatal Depression Scale; PD: postnatal depression; OR: odds ratios; COR: crude odds ratios; AOR: adjusted odds ratios.

## Competing interests

The authors declare that they have no competing interests.

## Authors' contributions

AZ and MTS administered the test and collected data. VR performed the statistical analysis. SM collected and interpreted data. MLM drafted and revised the manuscript. VC and EDR conceived the study, participated in its design and coordination and helped to draft the manuscript.

All authors read and approved the final manuscript.
